# Effects of the Combined Treatment of Trans-2-Hexenal, Ascorbic Acid, and Dimethyl Dicarbonate on the Quality in Fresh-Cut Potatoes (*Solanum tuberosum* L.) during Storage

**DOI:** 10.3390/foods13101526

**Published:** 2024-05-14

**Authors:** Yu Liu, Jiayi Zhang, Yaqin Zhao, Yinqiu Bao, Zhengguo Wu, Yonghua Zheng, Peng Jin

**Affiliations:** College of Food Science and Technology, Nanjing Agricultural University, Nanjing 210095, China; 2023208010@stu.njau.edu.cn (Y.L.); 2021808144@stu.njau.edu.cn (J.Z.); 2022208012@stu.njau.edu.cn (Y.Z.); 2021208011@stu.njau.edu.cn (Y.B.); wuzg@njau.edu.cn (Z.W.); zhengyh@njau.edu.cn (Y.Z.)

**Keywords:** fresh-cut potatoes, trans-2-hexenal, ascorbic acid, dimethyl dicarbonate, quality deterioration, reactive oxygen species, energy, γ-aminobutyric acid

## Abstract

Fresh-cut potatoes (*Solanum tuberosum* L.) are susceptible to browning and microbial contamination during storage. In this study, the effects of trans-2-hexenal (E2H), ascorbic acid (VC), dimethyl dicarbonate (DMDC), and the combined treatment of E2H, VC, and DMDC on quality deterioration in fresh-cut potatoes were investigated. The response surface methodology (RSM) demonstrated that E2H, VC, and DMDC concentrations of 0.010%, 0.65%, and 240 mg/L, respectively, were the optimum conditions for fresh-cut potato preservation. Further analysis showed that the combined treatment of E2H, VC, and DMDC was the most effective method of reducing quality deterioration in potatoes compared to the control and individual treatments. Furthermore, the combined treatment of E2H, VC, and DMDC could decrease the accumulation of reactive oxygen species (ROS) via improving antioxidant enzyme activities. Meanwhile, energy-metabolism-related enzyme activities and glutamate decarboxylase (GAD) activity were enhanced, while γ-aminobutyric acid transaminase (GABA-T) activity was reduced via the combined treatment of E2H, VC, and DMDC, which contributed to maintaining high energy levels and GABA content in potatoes. These findings suggested that the combined treatment of E2H, VC, and DMDC could protect membrane integrity through enhancing antioxidant capacity, energy levels, and GABA content to maintain quality in fresh-cut potatoes.

## 1. Introduction

In recent years, there has been a rapid increase in demand for fresh-cut fruits and vegetables due to their convenience [1,2]. Potatoes (*Solanum tuberosum* L.) are a highly nutritious food source, with high levels of starch, vitamins, and dietary fiber, which are gradually becoming a major product in the fresh-cut products industry [1]. Fresh-cut potatoes have been well-received by consumers, fast-food industries, and restaurants due to their freshness and convenience in the current fast-paced life [2]. However, after peeling and cutting, potatoes are susceptible to quality deterioration, such as surface browning and microbiological contamination, which commonly shortens the shelf life in fresh-cut potatoes [3]. Accordingly, there is a need to develop efficient techniques to mitigate browning and reduce microbiological contamination in fresh-cut potatoes.

Reactive oxygen species (ROS), such as hydrogen peroxide (H_2_O_2_) and superoxide radical (O_2_^•−^), are present at low levels under normal physiological conditions in postharvest horticultural crops but accumulate rapidly under stress conditions, such as mechanical damage, cold, and drought [4]. Excessive production of ROS results in the peroxidation of membrane lipids, disrupting cell membrane integrity and leading to browning in fruits and vegetables [5]. Increasingly, it has been shown that improving antioxidant enzyme activities, including superoxide dismutase (SOD), catalase (CAT), and ascorbate peroxidase (APX), was beneficial for inhibiting ROS accumulation, thereby mitigating browning in fresh-cut products [6]. In potatoes, it was demonstrated that the alleviation of surface browning was closely associated with high activities of SOD, CAT, and APX, which contributed to maintaining ROS homeostasis and protecting membrane structure [7]. In addition, a previous study also revealed that ultrasonic treatment attenuated browning in potatoes by improving antioxidant capacity through reducing the 1,1-diphenyl-2-picrylhydrazyl (DPPH) radical [8]. Thus, enhancing antioxidant capacity to reduce ROS overproduction is closely connected with alleviating surface browning in fresh-cut potatoes, and investigating its mechanism is crucial.

Cellular energy supply exhibits a considerable effect on ripening and senescence in crops, whereas insufficient energy supply may lead to physiological disorders in horticultural crops [9]. The lack of energy supply results in cell membrane susceptibility to damage and membrane structure instability, which can lead to the occurrence of browning [10]. Many studies have confirmed that maintaining high energy levels through improving the activity of enzymes involved in energy metabolism, such as succinic dehydrogenase (SDH), cytochrome C oxidase (CCO), H^+^-ATPase, and Ca^2+^-ATPase, could attenuate browning and prolong the storage time of fruits and vegetables [11,12]. In potatoes, it has been confirmed that high energy levels contributed to retarding the surface browning in potato slices [1]. Moreover, it has been reported that the increases in enzyme activities related to energy metabolism were able to effectively repress the pericarp browning in litchi fruit (*Litchi chinensis* Sonn.) [11] and surface browning in fresh-cut apples (*Malus domestica*) [12]. The above studies suggest that sufficient energy supply is essential for mitigating browning to maintain the quality of fresh-cut potatoes.

It is well known that γ-aminobutyric acid (GABA), an osmotic substance, is widely involved in various stress responses of postharvest horticultural crops [13]. The accumulation of GABA is conducive to maintaining cell osmotic balance and protecting subcellular structural integrity [14]. Many studies have proved that increasing glutamate decarboxylase (GAD) activity, a crucial enzyme in GABA synthesis, and depressing γ-aminobutyric acid transaminase (GABA-T) activity, a key enzyme for GABA catabolism, could improve GABA accumulation and subsequently reduce quality deterioration in fruits and vegetables [15,16,17]. Ali et al. [15] proposed that the enhancement of GABA content due to the regulation of GABA metabolism contributed to preventing the browning in aonla fruit (*Emblica officinalis* Gaertn.). Additionally, it has been confirmed that the alleviation of pericarp browning in pear fruit (*Pyrus ussuriensis* Maxim.) was attributed to the increase in GABA content by activating GAD activity, which might be owing to the decrease in the damage of membrane structure [16]. Furthermore, it has been confirmed that GABA treatment significantly reduced browning in potatoes [17]. These studies indicate that the modulation of GABA metabolism acts as a significant component in reducing quality deterioration in fresh-cut potatoes.

Trans-2-hexenal (E2H), a natural volatile, is formed and released in horticultural crops in response to biotic or abiotic stresses, exhibiting strong antibacterial activity against various microorganisms by damaging the cell membrane of pathogenic bacteria [18,19]. As a naturally occurring compound in plants, E2H has a high safety profile, and the US Food and Drug Administration has permitted it as an additive to foods [20]. Recently, E2H has been applied to preserve postharvest fruits, such as strawberry fruit (*Fragaria ananassa*) [21], tomato fruit (*Solanum lycopersicum*) [22], and pear fruit [20], which could repress the growth of microorganisms (bacteria and fungi) and maintain postharvest storage quality. Ascorbic acid (VC), as a kind of natural antioxidant, has been employed extensively in the inhibition of browning in fresh-cut products [23]. Furthermore, the combination of VC with other treatments like ultrasound and vacuum packaging has been demonstrated to be an efficient method of repressing browning in fresh-cut products [24,25]. Recently, the alleviated browning by VC combined with low-frequency ultrasound treatment, through protecting membrane integrity, has been proved in fresh-cut potatoes [24]. Dimethyl dicarbonate (DMDC), a food additive, is effective in inhibiting microbial growth (bacteria and fungi) due to its potential ability to bind to nucleophilic groups in microbial enzymes [26]. In fresh-cut carrots (*Daucus Carota* L.), it has been proved that DMDC treatment significantly reduced the growth of yeasts and molds and delayed the decreases in nutrition and quality in carrots [27]. Nonetheless, there is limited information on the impacts of the combined application of E2H, VC, and DMDC on the quality in fresh-cut potatoes and whether these effects are associated with the regulation of ROS, energy, and GABA metabolisms remains unclear.

Therefore, this study aimed to investigate the impacts of the combined treatment of E2H, VC, and DMDC on the quality in fresh-cut potatoes and to explore the metabolisms of ROS, energy, and GABA in order to clarify potential mechanisms of the combined treatment of E2H, VC, and DMDC causing a reduction in browning and microbiological contamination in fresh-cut potatoes. This study was able to provide a new technology in mitigating the quality deterioration of fresh-cut potatoes.

## 2. Materials and Methods

### 2.1. Material and Sample Preparation

Potatoes (*Solanum tuberosum* L. cv. ‘Netherlands 15′) with commercial maturity were obtained at a nearby vegetable store in Nanjing, China. Uniform-sized potatoes, devoid of physical damage, illness, pests, and germination, were selected at random and were initially washed with tap water, subsequently sanitized for 10 min using sodium hypochlorite (0.1%, *v*/*v*). After drying naturally, the potatoes were peeled and cut into 3–4 mm potato slices for subsequent experiments.

### 2.2. Design of Response Surface Methodology (RSM)

The Box–Behnken design (BBD) of RSM was applied to improve the effects of the variables on the quality of potatoes. The response surface experiments were designed with three factors, E2H concentration (*A*, 0.005–0.015%, *v*/*v*), VC concentration (*B*, 0.40–0.80%, *w*/*v*), and DMDC concentration (*C*, mg/L), as independent variables and L* value (*Y*_1_) and firmness (*Y*_2_) as response variables (Table 1). The ranges of E2H, VC, and DMDC concentrations were selected according to the preliminary single-factor experiments (Supplementary Tables S1–S3). The BBD design is listed in Supplementary Table S4, which contains 17 experimental runs. According to the 17 combination conditions, potato slices were soaked in various solutions for 5 min, while some potato slices were soaked in distilled water as the control group. Subsequently, potatoes were placed at 4 °C for 6 d. On day 6, L* value and firmness in potato slices were assayed.

### 2.3. The Combined Treatment of E2H, VC, and DMDC

Based on the optimum treatment conditions derived from RSM experiment results, fresh-cut potatoes were treated for further experiments. Prepared fresh-cut potatoes were assigned to five groups, each comprising three biological replicates. Fresh-cut potatoes were immersed in distilled water, 0.010% E2H solution, 0.65% VC solution, and 240 mg/L DMDC solution for 5 min as the control group, E2H-treated group, VC-treated group, and DMDC-treated group, respectively. For the E2H+VC+DMDC treatment group, fresh-cut potatoes were dipped in a solution containing 0.010% E2H, 0.65% VC, and 240 mg/L DMDC for 5 min. Afterward, potato slices were stored for 6 d at 4 °C. Samples were taken every day during storage, frozen in liquid nitrogen, and then, stored at −20 °C for subsequent measurements.

### 2.4. L*, a* and b* Values, Sensory Score, Total Mold and Yeast Count, and Firmness

The hand-held colorimeter (CR400, Konica, Japan) was applied to assay the color of potatoes in different treatment groups during storage. The values of L*, a*, and b* (brightness, red-greenness, and yellow-blueness) in potatoes were noted by measuring the middle part of each potato slice using the colorimeter. Three replicate experiments were performed for each group. The measurement of the sensory score was referenced to Feng et al. [23]. Potato slices were scored according to the following five grades: grade 1 (90–100 points), no browning; grade 2 (80–89 points), slight browning; grade 3 (70–79 points), moderate browning; grade 4 (60–69 points), moderately severe browning; grade 5 (below 60 points), severe browning. The limit of salability was considered to be a score of 70. On days 0, 1, 2, 3, 4, 5, and 6 of storage, potatoes were taken to score sensory quality. In addition, the assay of total mold and yeast count was in conformity with a previous study [28], and the result was denoted as log CFU/kg based on the fresh weight (FW). Moreover, the firmness was determined using a texture analyzer (TMS-PRO, Sterling, VA, USA) with the following parameters: probe diameter (5 mm), trigger force (2 N), puncture speed (60 mm/min), and puncture distance (1.5 mm); the result was denoted as N.

### 2.5. Determination of O_2_^•−^ Production Rate, H_2_O_2_ Content, and DPPH Radical Scavenging Rate

The methods for assaying O_2_^•−^ production rate, H_2_O_2_ content, and DPPH radical scavenging rate were referenced by Wang et al. [29]. A frozen sample (1 g) was taken and ground with phosphate buffer (Yuanye Bio-Technology Co., Ltd., Shanghai, China), followed by centrifugation, and a supernatant was taken for the O_2_^•−^ production rate assay. According to the standard curve of potassium nitrite (Macklin Biochemical Co., Ltd., Shanghai, China) measured by the spectrophotometer (Metash Instruments Co., Ltd., Shanghai, China) at 530 nm, the O_2_^•−^ production rate was calculated and represented as nmol/min/kg. For H_2_O_2_ content, 1 g of frozen sample was taken, ground with trichloroacetic acid solution (5 mL), and centrifuged, followed by mixing the supernatant with potassium iodide solution and phosphate buffer for 1 h. According to the standard curve measured by the spectrophotometer at 390 nm, the H_2_O_2_ content was calculated and denoted as μmol/kg. Moreover, the frozen sample (1 g) was taken and ground with methanol (Yuanye Bio-Technology Co., Ltd., Shanghai, China) followed by centrifugation, and the supernatant was taken for the scavenging rate of DPPH radical assay. Then, 0.1 mL of supernatant was mixed with 1.9 mL of DPPH solution (120 μmol/L). After 20 min of reaction, the absorbance value of the solution was measured at 517 nm. DPPH radical scavenging rate was expressed as a percentage.

### 2.6. Measurement of ROS Metabolism-Related Enzyme Activities

SOD, CAT, and APX activities were measured in agreement with the method of Fan et al. [30]. The method of nitro blue tetrazolium (NBT) photoreduction was utilized to assay SOD activity at 560 nm by the spectrophotometer. Five g of frozen sample was taken, ground with phosphate buffer (5 mL), and centrifuged, followed by mixing the supernatant with 750 μmol/L NBT, 130 mmol/L methionine, and 20 μmol/L riboflavin for 15 min. Finally, the absorbance value of the solution was measured at 560 nm. The quantity of the enzyme that inhibited 50% of the NBT reduction was considered to be one unit (U) of SOD activity. Additionally, the frozen sample (1 g) was taken and ground with phosphate buffer followed by centrifugation, and the supernatant was taken for the CAT and APX activities assay. For CAT activity assay, 0.1 mL of the supernatant was mixed with 1.9 mL of H_2_O_2_ solution (20 mmoL/L). After 3 min of reaction, the absorbance value of the solution was measured at 240 nm. One U of CAT activity was considered as a 0.01 change in absorbance per minute. In addition, for the APX activity assay, 0.1 mL of the supernatant was mixed with 1.9 mL of H_2_O_2_ solution (20 mmoL/L). After 3 min of reaction, the absorbance value of the solution was measured at 290 nm. One U of APX activity referred to a 0.01 change in absorbance per minute. The results were denoted as U/kg.

### 2.7. Assay of ATP, ADP, and AMP Contents, and Energy Charge

The ATP, ADP, and AMP contents were detected based on the approach of Li et al. [10]. The frozen sample was ground in perchloric acid (5 mL, 0.6 M, Yuanye Bio-Technology Co., Ltd., Shanghai, China), then centrifuged (Anting Scientific Instrument, Co., Ltd., Shanghai, China) at 12,000× *g*. Following that, the supernatant (3 mL) was removed, and using 1 M potassium hydroxide solution (Yuanye Bio-Technology Co., Ltd., Shanghai, China), its pH was adjusted to 6.5–6.8 before being diluted to 4 mL using distilled water. After standing for 30 min, the solution was filtered by a membrane filter (0.45 μm, Solarbio, Beijing, China) for further measurement. Subsequently, a high-performance liquid chromatography system (HPLC, Agilent 1100, Santa Clara, CA, USA) with a Nova-Pak C18 column (5 × 250 mm, 5 μm, Santa Clara, CA, USA) was applied to assay ATP, ADP, and AMP contents at a flow rate of 0.8 mL/min. In addition, mobile phase 1 and mobile phase 2 were potassium phosphate buffer (Yuanye Bio-Technology Co., Ltd., Shanghai, China) and pure methanol (Aladdin Bio-Chem Technology Co., Ltd., Shanghai, China), respectively. The elution was performed via a gradient elution procedure with 100%, 80%, and 100% of mobile phase 1 at 0, 7, and 10 min, respectively. The contents of ATP, ADP, and AMP were quantified by standard curves and represented as g/kg. Moreover, the formula below was used to assess the energy charge:

Energy Charge = (ATP + 0.5 × ADP)/(ATP + ADP + AMP)(1)

### 2.8. Determination of Energy-Metabolism-Related Enzyme Activities

The measurement of enzyme activities related to energy metabolism was in conformity with a previous study [31]. The frozen sample (5 g) was ground and filtered through gauze before centrifuging. Then, the precipitate was centrifuged again after being washed with Tris-HCl (10 mM, pH 7.2). The mitochondrial enzyme solution for assaying energy-metabolism-related enzyme activities was obtained after adding 1.5 mL of washing solution to the final precipitate. One U of H^+^-ATPase or Ca^2+^-ATPase activity referred to 1 μmol/L of inorganic phosphorus released at 660 nm per min. Furthermore, a change in absorbance of 0.01 at 600 nm or 510 nm per min was considered as 1 U of SDH or CCO activity, respectively. The results were denoted as U/kg.

### 2.9. Measurement of GABA Content, GAD, and GABA-T Activity

The GABA content and GAD activity were assayed following the approach of Liu et al. [13]. For the determination of GABA content, the frozen sample was ground in lanthanum chloride solution (5 mL, 0.05 M) and then centrifuged. Subsequently, the supernatant (2 mL) was combined with KOH solution (0.5 mL, 1 M) and mixed at 28 °C. After that, the solution was combined with KOH solution, sodium hypochlorite solution, boric acid-borax buffer, and phenol solution, then added with 0.8 mL of 60% ethanol solution in a boiling water bath for 10 min and, next, in an ice bath for 5 min. Finally, the GABA content was assessed via a standard curve and expressed as g/kg. One U of GAD activity was considered equivalent to the production of 1 μg of GABA per h. In addition, GABA-T activity was measured applying the ELISA kit (Jiangsu Meimian Industrial Co., Ltd., Yancheng, China) on the basis of the manufacturer’s instructions. The results of GAD and GABA-T activities were denoted as U/kg.

### 2.10. Statistical Analysis

Each experiment was conducted in triplicate, and all data were expressed as means ± standard errors (SE). All data were analyzed using the one-way analysis of variance (ANOVA) with IBM SPSS Statistics 26. Additionally, means were compared by Duncan’s multiple range tests, and *p* < 0.05 was regarded statistically significant. Design Expert 8.0.6 software was applied for RSM design, model fitting, and plotting of response surface curves.

## 3. Results

### 3.1. BBD Model Fitting and Statistical Analysis

As shown in the results of the single-factor experiments in Supplementary Tables S1–S3, in comparison with the control group, L* value and firmness were highest in the 0.010% E2H-treated group on days 3 and 6 of storage. On days 3 and 6 of storage, the L* value of the 0.60% VC-treated group was remarkably higher than the other groups. In addition, L* value and firmness of the control were remarkably lower than those in 250 mg/L DMDC-treated group on day 6 of storage. Therefore, 0.010% E2H, 0.60% VC, 250 mg/L DMDC were chosen as the central points of the response surface test.

The L* value and firmness in potatoes of various groups were measured on day 6 of storage (Supplementary Table S4). Furthermore, multinomial regression analysis was performed to obtain two fitted quadratic multinomial regression equation models, and the relationship between the response values (*Y*_1_ and *Y*_2_) and the independent variables (*A*, *B*, and *C*) was expressed by the following quadratic multiple regression equations:

*Y*_1_ = 66.50 + 0.034*A* + 4.83*B −* 1.76*C* − 1.13*AB* + 3.03*AC* + 0.26*BC* − 4.28*A*^2^ − 3.92*B*^2^ − 4.71*C*^2^ (2)

*Y*_2_ = 37.46 + 0.42*A* − 0.12*B* − 1.49*C* + 0.42*AB* + 0.71*AC* + 0.66*BC* − 2.19*A*^2^ − 1.47*B*^2^ − 2.49*C*^2^ (3)

As shown in Supplementary Table S5, the L* value and firmness models were significant (*p* < 0.05). The coefficient of determination R^2^ > 0.83, and the model-adjusted coefficient of determination R_adj_^2^ in L* value and firmness models were 0.8944 and 0.6242, respectively, implying that the models were dependable and that the experimental error was small. Furthermore, the model was fit by the experimental data, as evidenced by the *p*-values of the lack of fit in L* value and firmness models, which were 0.1081 and 0.1584 (*p* > 0.05, not significant), respectively.

### 3.2. Analysis of Response Surface Interaction

**Figure 1:**
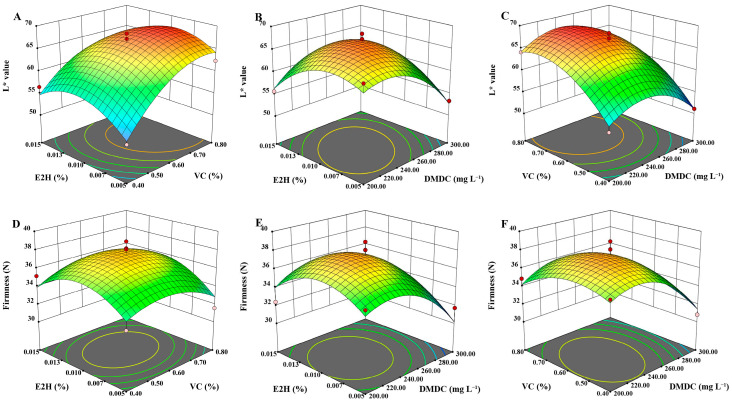
Response surface graphs exhibit effects of treatment variables on (**A**–**C**) L* value and (**D**–**F**) firmness in potatoes.

In Figure 1, the response surface plots show the effects of different independent factors on the L* value and firmness of potatoes. When the concentration of DMDC was 250 mg/L and the concentration of VC was in the range of 0.60% to 0.70%, the higher L* value was observed (Figure 1A). The L* value in potatoes was discovered to be lower when the concentration of VC was fixed at 0.60%, and the more the E2H concentration and DMDC concentration departed from the central level, implying that the interaction between these two variables had a significant impact on the L* value (*p* < 0.05) (Figure 1B). In addition, when the concentration of E2H was fixed at 0.010%, the more DMDC concentration departed from the central level, and the faster the L* value decreased (Figure 1C). The interaction effects of different factors in firmness are shown in Figure 1D–F. Lower firmness was noted as the E2H and VC concentrations departed more from the center level at a DMDC concentration of 250 mg/L. Moreover, when VC concentration was fixed at 0.60%, the more DMDC concentration deviated from the central level, the surface of the firmness model became steeper. The VC concentration between 0.60% and 0.70% and the concentration of DMDC at about 240 mg/L could maintain a higher firmness in potatoes.

The optimum treatment conditions analyzed by RSM for maintaining the good quality of potatoes were 0.010% E2H, 0.65% VC, and 238.58 mg/L DMDC. Considering the operational feasibility, the DMDC concentration was changed to 240 mg/L. Consequently, the E2H concentration of 0.010%, the VC concentration of 0.65%, and the DMDC concentration of 240 mg/L were chosen for subsequent validation experiments to confirm and assess the reliability and validity of the model. The results showed that E2H+VC+DMDC treatment significantly maintained a higher L* value and firmness in potatoes than the other groups on day 6 of storage (Supplementary Table S6), suggesting that the combined treatment of E2H, VC, and DMDC was the valid method for maintaining the good quality of potatoes.

### 3.3. Quality Changes in Fresh-Cut Potatoes

**Figure 2:**
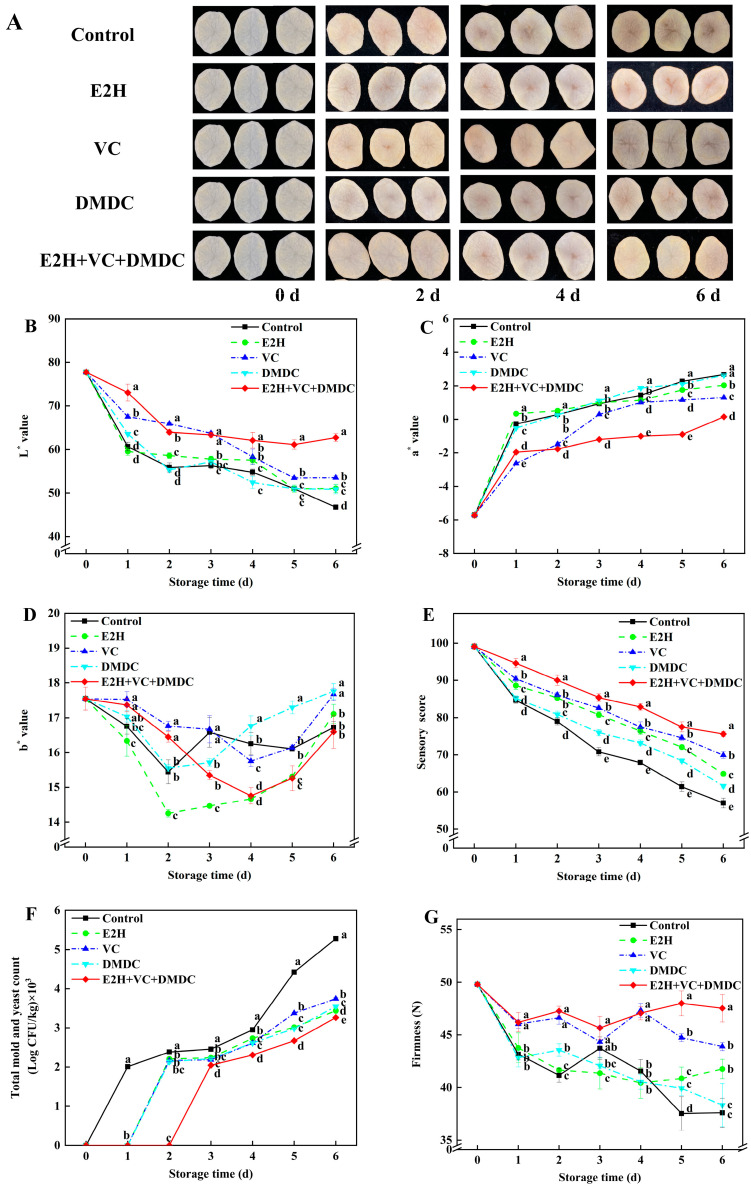
The combined treatment of E2H, VC, and DMDC maintained the quality of potatoes. (**A**) The visual appearance of potatoes during storage. The variations in potatoes during storage in terms of (**B**) L* value, (**C**) a* value, (**D**) b* value, (**E**) sensory score, (**F**) total mold and yeast count, and (**G**) firmness. Vertical bars indicate the SE of triplicate assays. Different letters indicate significant differences (*p* < 0.05).

Figure 2A shows the changes in appearance quality of potatoes during storage. Slight browning began to appear in the center of potato slices on the second day of storage, and the degree of browning deepened as the storage time extended. Compared to the control, E2H, VC, and DMDC groups, the combined treatment of E2H, VC, and DMDC significantly alleviated the browning of potatoes. The L* value and sensory score of control potatoes declined continuously, whereas the combined treatment of E2H, VC, and DMDC markedly repressed their decreases throughout the whole storage time (Figure 2B,E). On day 6, the L* value and sensory score of potatoes in the E2H+VC+DMDC treatment group were 62.74 and 75.60, respectively, which were 34.26% and 32.63% higher than those in control. Additionally, the a* value and total mold and yeast count in potatoes rose constantly during storage and reached peak level by the end of storage (Figure 2C,F). The a* value in the control potatoes was −5.71 at the start of storage but reached a maximum on day 6 at 2.67. However, the a* value in E2H+VC+DMDC-treated potatoes was 0.15 on day 6 of storage, which was the lowest compared with the other four groups. Total mold and yeast count in E2H+VC+DMDC-treated potatoes maintained the lowest level compared with the other four groups during the entire storage time. Moreover, the b* value in E2H+VC+DMDC-treated potatoes decreased constantly until day 4 of storage and increased thereafter (Figure 2D). On day 4, the b* value in E2H+VC+DMDC-treated potatoes was 14.76, which was 9.17% lower than those in control, displaying a significant difference (*p* < 0.05). Moreover, the firmness in E2H+VC+DMDC-treated potatoes remained at a higher level throughout the entire storage time compared with the control (Figure 2G). On day 6, the firmness of potatoes treated with E2H+VC+DMDC was 26.41% higher than those in control, exhibiting a significant difference.

### 3.4. O_2_^•−^ Production Rate, H_2_O_2_ Content, DPPH Radical Scavenging Rate, and Activities of ROS Metabolism Enzymes

**Figure 3:**
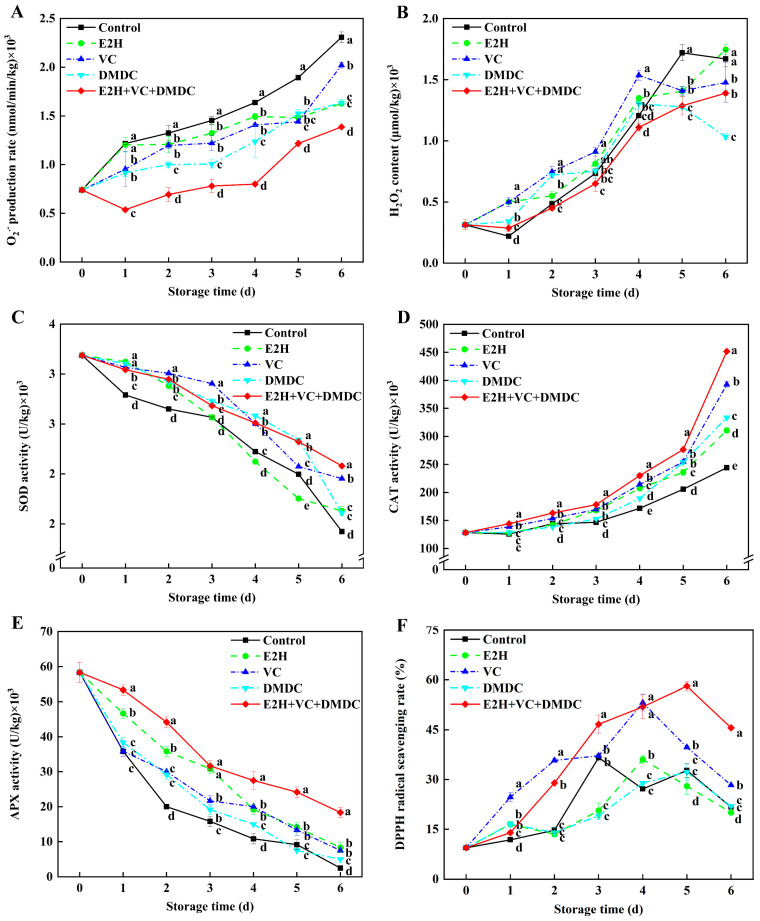
The combined treatment of E2H, VC, and DMDC modulated ROS metabolism in potatoes. The variations in potatoes during storage in terms of (**A**) O_2_^•−^ production rate, (**B**) H_2_O_2_ content, (**C**) SOD activity, (**D**) CAT activity, (**E**) APX activity, and (**F**) DPPH radical scavenging rate. Vertical bars indicate the SE of triplicate assays. Different letters indicate significant differences (*p* < 0.05).

O_2_^•−^ production rate and H_2_O_2_ content in E2H+VC+DMDC-treated potatoes reduced during the first day of storage and, then, rose gradually (Figure 3A,B). All of the E2H, VC, DMDC, and E2H+VC+DMDC treatments were efficient in reducing the O_2_^•−^ production rate compared with the control. In contrast to the other four groups, E2H+VC+DMDC treatment significantly decreased the O_2_^•−^ production rate, which was 39.83% lower than that of the control group on the last day of storage. Moreover, the potatoes treated with E2H+VC+DMDC maintained a lower H_2_O_2_ content after 2 days of storage than the control. The DPPH radical scavenging rate of potatoes in the E2H+VC+DMDC treatment group peaked on day 5 of storage and descended thereafter (Figure 3F), which was remarkably higher than that of the other four groups on the last day of storage.

SOD and APX activities in potatoes exhibited a similar tendency, which declined constantly until day 6 of storage, while the activity of CAT increased continuously throughout the entire storage time (Figure 3C–E). In contrast to the control, the activity of SOD in E2H+VC+DMDC-treated potatoes remained at a higher level throughout the entire storage time. On day 6, the SOD activity of potatoes treated with E2H+VC+DMDC peaked at 46.48% higher than those in control, exhibiting a significant difference (*p* < 0.05). Additionally, all treatments enhanced CAT and APX activities compared to the control during storage. However, the combined treatment of E2H, VC, and DMDC was more efficient in improving CAT and APX activities than the individual E2H, VC, or DMDC treatment. These findings suggested that the combined treatment of E2H, VC, and DMDC reduced the accumulation of ROS in fresh-cut potatoes by increasing SOD, CAT, and APX activities.

### 3.5. ATP, ADP and AMP Contents, and Energy Charge

**Figure 4:**
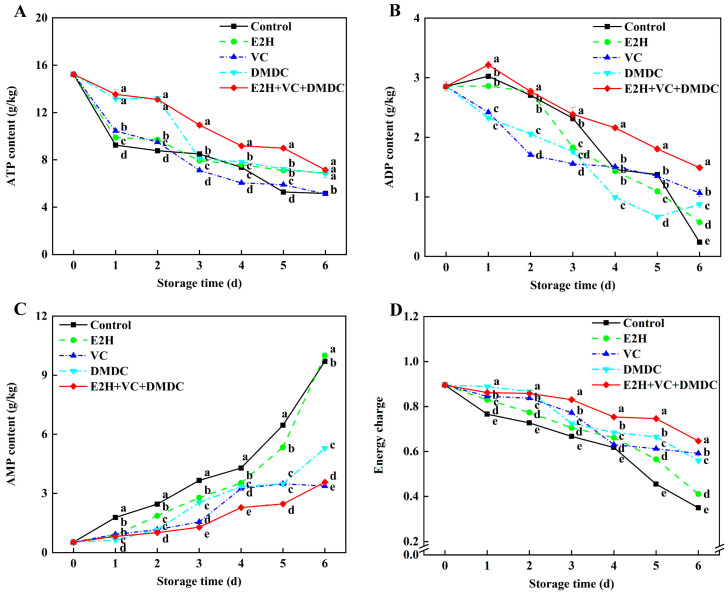
The combined treatment of E2H, VC, and DMDC modulated substance contents of energy metabolism in potatoes. The variations in potatoes during storage in terms of (**A**) ATP content, (**B**) ADP content, (**C**) AMP content, and (**D**) energy charge. Vertical bars indicate the SE of triplicate assays. Different letters indicate significant differences (*p* < 0.05).

ATP content in E2H+VC+DMDC-treated potatoes reduced continually during the entire storage period, whereas the ADP content of potatoes in E2H+VC+DMDC treatment group reached the peak on the first day of storage and descended afterward (Figure 4A,B). The E2H+VC+DMDC treatment group showed the highest ATP and ADP contents in potatoes during storage compared to the other four groups. On day 6, the ATP and ADP contents in E2H+VC+DMDC-treated potatoes were 38.43% and 520.83% higher than those in control. In addition, the AMP content and energy charge of potatoes in the control group rose and declined constantly during the whole storage time, respectively, while E2H+VC+DMDC treatment markedly deferred the enhancement of AMP content and the decline of energy charge (Figure 4C,D). All of the E2H, VC, DMDC, and E2H+VC+DMDC treatments maintained a higher energy charge than the control group, but the highest energy charge level was shown in E2H+VC+DMDC-treated potatoes after 3 days of storage.

### 3.6. Activities of Energy Metabolism Enzyme

**Figure 5:**
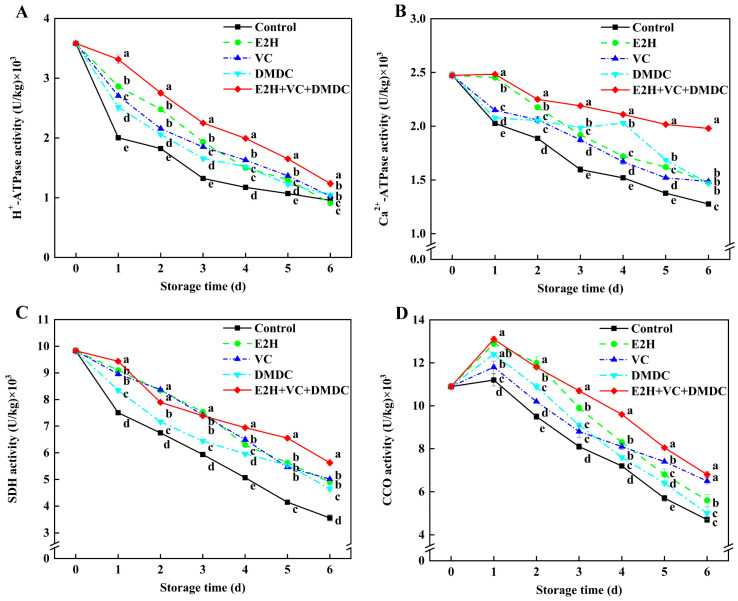
The combined treatment of E2H, VC, and DMDC modulated enzyme activities of energy metabolism in potatoes. The variations in potatoes during storage in terms of (**A**) H^+^-ATPase, (**B**) Ca^2+^-ATPase, (**C**) SDH, and (**D**) CCO activities. Vertical bars indicate the SE of triplicate assays. Different letters indicate significant differences (*p* < 0.05).

The activities of H^+^-ATPase, Ca^2+^-ATPase, and SDH in E2H+VC+DMDC-treated potatoes declined continuously until the end of storage, while CCO activity rose early in the storage period and dropped afterwards (Figure 5). Compared to the control, E2H+VC+DMDC treatment significantly enhanced H^+^-ATPase, Ca^2+^-ATPase, and SDH activities that were 29.17%, 54.69%, and 58.15% higher than those in control on the last day of storage, respectively. Additionally, CCO activity in E2H+VC+DMDC-treated potatoes peaked on the first day of storage compared to the other four groups, which was 16.96%, 1.55%, 11.02%, and 5.65% higher than those in control, E2H, VC, and DMDC groups, respectively. The above results showed that the combined treatment of E2H, VC, and DMDC improved energy levels in fresh-cut potatoes through activating enzyme activities involved in energy metabolism.

### 3.7. GABA Content, GAD, and GABA-T Activities

**Figure 6:**
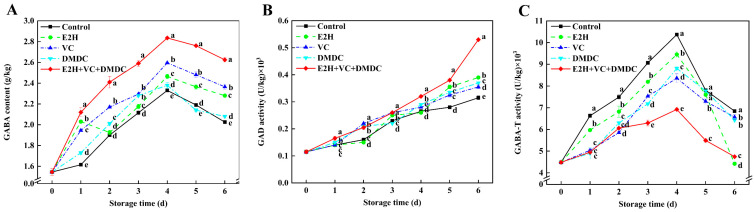
The treatment of combined E2H, VC, and DMDC modulated GABA metabolism in potatoes. The variations in potatoes during storage in terms of (**A**) GABA content, (**B**) GAD, and (**C**) GABA-T activities. Vertical bars indicate the SE of triplicate assays. Different letters indicate significant differences (*p* < 0.05).

In Figure 6A, the GABA content of potatoes in the control peaked on day 4 of storage and reduced thereafter. Compared with the control, E2H, VC, and DMDC treatments, the E2H+VC+DMDC treatment kept a significantly higher level of GABA during the entire storage time. Moreover, the GAD activity of potatoes rose gradually until day 6 of storage (Figure 6B). At the end of storage, GAD activity in E2H+VC+DMDC-treated potatoes was noticeably enhanced, which was 68.25% higher than that of the control. The GABA-T activity of potatoes rose early in the storage time and decreased afterwards, and the E2H+VC+DMDC-treated group exhibited the lowest GABA-T activity in potatoes than the other four groups on day 4 of storage (Figure 6C), which was 33.27% lower than that in the control group. The findings implied that E2H+VC+DMDC treatment enhanced GABA content in potatoes by increasing GAD activity and suppressing GABA-T activity.

## 4. Discussion

While fresh-cut products are preferred by consumers because of the convenience for cooking, they are susceptible to browning and microbial growth on account of the mechanical damage resulting from the cutting process, leading to a reduction in sensory quality and commercial value [32]. Therefore, the search for effective techniques to alleviate browning and inhibit the growth of microorganisms in potatoes has become a major concern. Many studies have confirmed that the combined treatment was more efficient in maintaining the quality of fresh-cut products than individual treatments [25,33]. In addition, the use of RSM analysis to optimize combined treatment conditions to maintain the quality of fresh-cut products has been demonstrated nowadays on fresh-cut oranges [34] and fresh-cut apples [35]. In this study, the optimum condition of the combined treatment of E2H, VC, and DMDC (0.010% E2H, 0.65% VC, and 240 mg/L DMDC) which effectively inhibited the browning and maintained the good quality of potatoes was determined by RSM. Subsequently, the microbial growth and the browning degree of potatoes were assessed under the optimal treatment condition. L*, a*, and b* values, representing the color change, are considered to be indicators that indicate surface browning severity in potato slices [36]. In this study, the E2H+VC+DMDC treatment significantly alleviated surface browning, maintained high levels of L* value, sensory score, and firmness, and decreased a* value, b* value, and total mold and yeast count in potatoes (Figure 2), suggesting that E2H+VC+DMDC treatment was instrumental in mitigating browning and suppressing microbial growth in potatoes. E2H treatment has been shown to be a valid method for inhibiting microbial decay in strawberry fruit [21] and tomato fruit [22]. Xu et al. [24] also found that VC treatment was capable of alleviating surface browning in potatoes. Furthermore, in fresh-cut carrots, it was also shown that the decrease in microbial growth and the maintenance of good quality in carrots were due to the application of DMDC [27].

The homeostasis between the generation and elimination of ROS in postharvest horticultural crops can be disrupted under diverse biotic and abiotic stresses, leading to excessive accumulation of ROS regulated by enzymes like SOD, CAT, and APX [3]. Moreover, membrane stability is reduced due to membrane lipid peroxidation caused by the overproduction of ROS [30]. Once the membrane structure is disrupted, it exacerbates the enzymatic reaction between phenolics and polyphenol oxidase, resulting in browning [2]. Many studies have proved that the enhancement of antioxidant capacity to reduce ROS accumulation is beneficial in maintaining cell membrane stability, thus reducing the surface browning caused by mechanical damage in fresh-cut products [37,38]. Ding et al. [37] reported that the improvement of quality in red pitaya fruit treated by acid electrolytic water was due to the low levels of O_2_^•−^ production rate and H_2_O_2_ content caused by increasing SOD, CAT, APX activities. In fresh-cut stem lettuce, the surface browning was mitigated by increasing CAT activity and decreasing O_2_^•−^ production rate and H_2_O_2_ content to maintain ROS homeostasis [38]. In this study, E2H+VC+DMDC treatment could remarkably improve SOD, CAT, and APX activities, increase DPPH radical scavenging rate, and repress the rise of O_2_^•−^ production rate and H_2_O_2_ content in potatoes (Figure 3). The above results correlated with the lighter surface browning in E2H+VC+DMDC-treated potatoes, suggesting that improved antioxidant capability could repress ROS accumulation and thus suppress membrane damage to attenuate surface browning of potatoes. Similar results were also confirmed in fresh-cut potatoes treated with GABA [17], proline [2], and tea polyphenols [6]. You et al. [1] reported that chlorogenic acid alleviated browning in potato slices via increasing SOD, CAT, and APX activities to reduce O_2_^•−^ production rate, H_2_O_2_ content, and DPPH radical content. Therefore, it could be concluded that E2H+VC+DMDC treatment maintained the quality in fresh-cut potatoes via activating antioxidant enzyme activities to suppress ROS increase and protect membrane integrity.

It is widely recognized that insufficient cellular energy supply, resulting from the disruption of mitochondrial structure and function, accelerates senescence and quality deterioration of postharvest horticultural crops [31]. So as to respond to the mechanical damage due to fresh-cut processing, sufficient energy must be provided to control the overproduction of ROS and maintain membrane stability to mitigate the browning of fresh-cut products [12]. Several enzymes, including H^+^-ATPase, Ca^2+^-ATPase, CCO, and SDH, are essential enzymes in the mitochondria to regulate energy status [31]. ATPases are able to convert ATP to ADP and release energy used to maintain the physiological activities of fruits and vegetables [39]. SDH can catalyze the dehydrogenation of succinic acid to produce fumaric acid and enable the electron transfer of hydrogen ions to form ATP, while CCO is the final enzyme of the electron transport chain of mitochondrial respiration and is also closely related to ATP production [40]. In our study, E2H+VC+DMDC treatment could significantly increase ATP content, ADP content, and energy charge and reduce AMP content as the results of the increases in H^+^-ATPase, Ca^2+^-ATPase, SDH, and CCO activities in potatoes (Figure 4 and Figure 5), which might be associated with retaining membrane integrity to attenuate the browning of potatoes. The previous work also found that the enhancement of enzyme activities related to energy metabolism by chlorogenic acid treatment maintained the high levels of energy charge and ATP and ADP contents, which were conducive to the alleviation of surface browning in potato slices [1]. Wu et al. [39] reported that higher H^+^-ATPase and Ca^2+^-ATPase activities were beneficial for improving the resistance of fresh-cut melons to cut-wounding stress. Chen et al. [12] also confirmed that the up-regulated expression of genes in relation to energy-metabolism-related enzymes might enhance ATP synthesis, thereby providing sufficient energy to maintain cell membrane structure for mitigating browning in fresh-cut apples. Taken together, it could be inferred that the attenuation of surface browning in E2H+VC+DMDC-treated potatoes was due to the maintenance of membrane structure resulting from increased energy levels through activating the activities of energy-metabolism-related enzymes.

Several studies have demonstrated that GABA content accumulates in abundance in response to abiotic stresses such as mechanical damage, low temperature, and drought to improve resistance in plants to adversity [41]. The primary pathway for GABA production, known as the GABA shunt, involves two key enzymes for GABA synthesis and catabolism. GAD enables catalysis of the production of GABA from glutamate, and GABA-T participates in the catabolism of GABA [16]. In addition, it has been demonstrated that regulating enzyme activities involved in GABA metabolism can promote the increase in GABA content, which contributes to maintaining membrane stability and suppressing browning in loquat fruit [13] and fresh-cut pear fruit [42]. Liu et al. [13] confirmed that the internal browning in PSKα-treated loquat fruit was remarkably mitigated because of the high level of GABA via increasing GAD activity and suppressing GABA-T activity, which might correlate with the integrity of membrane structure. In fresh-cut pear fruit, carbon dioxide treatment could maintain membrane structure through inducing GABA accumulation to alleviate the browning of pears [42]. In our current study, E2H+VC+DMDC treatment significantly activated GAD activity and decreased GABA-T activity, contributing to the enhancement of GABA content in potatoes (Figure 6), indicating that high GABA content resulting from modulating GAD and GABA-T activities were conducive to attenuating surface browning in potatoes.

Among all treatment groups, the E2H+VC+DMDC treatment group exhibited the highest antioxidant capacity, energy levels, and GABA content compared to the other four groups, which was advantageous to attenuate membrane lipid peroxidation and maintain membrane stability. Furthermore, the advantage of the combined treatment of E2H, VC, and DMDC in reducing the quality deterioration in fresh-cut potatoes might be owing to the additive or even synergistic impacts of E2H, VC, and DMDC treatments. Consequently, the improvement of sensory quality in E2H+VC+DMDC-treated fresh-cut potatoes during storage might be a consequence of the maintenance of ROS homeostasis and higher levels of ATP and GABA, which are beneficial for protecting the integrity of the membrane structure.

## 5. Conclusions

In conclusion, the combined treatment of E2H, VC, and DMDC effectively reduced the quality deterioration in fresh-cut potatoes during storage, including mitigating surface browning and inhibiting the growth of microorganisms. The combined treatment of E2H, VC, and DMDC eliminated ROS overproduction by activating SOD, CAT, and APX activities, resulting in repressing membrane lipid peroxidation. Moreover, the maintenance of quality in potatoes via the combined treatment of E2H, VC, and DMDC was associated with the high energy levels and GABA content resulting from modulating energy and GABA metabolisms, which might contribute to protecting membrane structure. These findings indicate that the combination of E2H, VC, and DMDC application would be a promising technology to extend the shelf life of fresh-cut potatoes.

## Figures and Tables

**Table 1:** Coded and levels of independent variables in RSM.

Variables	Coded	Levels	Independent Levels		
			−1	0	+1
E2H (%)	A	*x*_1_	0.005	0.010	0.015
VC (%)	B	*x*_2_	0.40	0.60	0.80
DMDC (mg/L)	C	*x*_3_	200	250	300

## Data Availability

The original contributions presented in the study are included in the article/Supplementary Material, further inquiries can be directed to the corresponding author.

## Supplementary Materials

The following supporting information can be downloaded at: https://www.mdpi.com/article/10.3390/foods13101526/s1, Table S1: Effect of E2H treatment on quality of fresh-cut potatoes during storage; Table S2: Effect of VC treatment on quality of fresh-cut potatoes during storage; Table S3: Effect of DMDC treatment on quality of fresh-cut potatoes during storage; Table S4: The experimental design and results; Table S5: Analysis of variance for regression equation; Table S6: Results of validation experiments for BBD.
