# Supplementary material for: Effects of the Combined Treatment of Trans-2-Hexenal, Ascorbic Acid, and Dimethyl Dicarbonate on the Quality in Fresh-Cut Potatoes (Solanum tuberosum L.) during Storage
Source: Foods. 2024 May 14;13(10):1526. doi: 10.3390/foods13101526 (PMC11120313; doi:10.3390/foods13101526)
Supplement: Supplementary file 1 [file foods-13-01526-s001.zip › foods-2968832-supplementary.pdf]

Supplementary Materials

Table S1. Effect of E2H treatment on quality of fresh-cut potatoes during storage.

Storage time (d)	Concentration (%) (v/v)	L* value	Firmness (N)
0		70.23±0.14	41.43±0.42
3	Control	48.98±0.38^{d}	32.53±0.15^{c}
	0.005	55.97±0.63^{b}	38.23±2.87^{b}
	0.010	61.27±0.81^{a}	42.87±0.83^{a}
	0.015	53.59±0.97^{c}	38.87±2.73^{b}
	0.020	51.52±1.63^{c}	38.33±0.27^{b}
	0.025	46.00±0.64^{e}	37.63±0.67^{b}
6	Control	44.83±0.24^{e}	30.97±1.03^{d}
	0.005	48.83±0.38^{c}	34.60±0.26^{b}
	0.010	58.94±0.74^{a}	37.43±0.23^{a}
	0.015	50.97±0.19^{b}	33.23±0.31^{c}
	0.020	46.29±0.41^{d}	31.33±0.06^{d}
	0.025	41.98±0.27^{f}	31.00±0.36^{d}

Results are expressed as mean±SE of triplicate assays. Different letters in a column imply statistically significant differences at the $p < 0.05$ level between control and treated groups according to Duncan's multiple range test.

Table S2. Effect of VC treatment on quality of fresh-cut potatoes during storage.

Storage time (d)	Concentration (%) (w/v)	L* value	Firmness (N)
0		70.23±0.14	41.43±0.42
3	Control	48.98±0.38^{d}	32.53±0.15^{d}
	0.10	60.09±0.40^{c}	40.57±1.47^{c}
	0.20	60.80±0.77^{c}	41.03±0.43^{c}
	0.40	61.15±0.06^{c}	41.77±1.47^{c}
	0.60	66.30±1.36^{a}	52.43±1.33^{a}
	0.80	62.98±0.08^{b}	47.17±0.47^{b}
6	Control	44.83±0.24^{c}	30.97±1.03^{d}
	0.10	56.15±0.88^{b}	32.53±0.15^{d}
	0.20	56.71±0.65^{b}	31.13±0.06^{d}
	0.40	56.89±0.68^{b}	33.23±0.12^{c}
	0.60	62.80±1.71^{a}	34.13±0.15^{b}
	0.80	56.86±0.05^{b}	36.83±0.38^{a}

Results are expressed as mean±SE of triplicate assays. Different letters in a column imply statistically significant differences at the $p < 0.05$ level between control and treated groups according to Duncan's multiple range test.

Table S3. Effect of DMDC treatment on quality of fresh-cut potatoes during storage.

Storage time (d)	Concentration (mg/L)	L* value	Firmness (N)
0		70.23±0.14	41.43±0.42
3	Control	48.98±0.38[e]	32.53±0.15[d]
	100	53.44±0.54[d]	37.07±0.43[b]
	150	54.55±0.12[c]	35.63±1.43[c]
	200	60.46±0.44[b]	38.03±0.73[b]
	250	62.93±0.33[a]	40.63±0.07[a]
	300	60.52±0.53[b]	41.57±0.63[a]
6	Control	44.83±0.24[e]	30.97±1.03[c]
	100	51.22±1.96[b]	32.57±0.21[b]
	150	53.64±1.14[c]	30.47±0.38[c]
	200	56.41±0.77[b]	32.87±0.15[b]
	250	61.12±0.89[a]	36.73±0.15[a]
	300	57.72±0.18[b]	27.20±0.44[a]

Results are expressed as mean±SE of triplicate assays. Different letters in a column imply statistically significant differences at the $p < 0.05$ level between control and treated groups according to Duncan's multiple range test.

Table S4. The experimental design and results.

Run	A: E2H (%)	B: VC (%)	C: DMDC (mg/L)	Y_1 L* value	Y_2 Firmness (N)
1	0.010	0.80	200.00	64.14	34.87
2	0.010	0.40	200.00	53.82	36.07
3	0.015	0.60	300.00	56.91	31.80
4	0.010	0.60	250.00	68.37	38.93
5	0.010	0.60	250.00	65.90	35.9
6	0.005	0.60	300.00	53.29	31.73
7	0.015	0.80	250.00	62.7	35.43
8	0.010	0.60	250.00	65.51	37.40
9	0.005	0.60	200.00	64.16	35.17
10	0.010	0.60	250.00	67.19	38.07
11	0.005	0.80	250.00	62.38	31.57
12	0.005	0.40	250.00	51.63	33.00
13	0.010	0.40	300.00	51.08	30.80
14	0.015	0.60	200.00	55.65	32.40
15	0.010	0.80	300.00	62.44	32.23
16	0.010	0.60	250.00	65.51	37.00
17	0.015	0.40	250.00	56.47	35.17

Results are expressed as mean±SE of triplicate assays.

Table S5. Analysis of variance for regression equation.

Source	L* value (Y_1)			Firmness (Y_2)		
	F-value	*p*-value	Significant	*F*-value	*p*-value	Significant
Model	16.05	0.0007	Significant	3.95	0.0418	Significant
A: E2H	0.0025	0.9611		0.5767	0.4724	
B: VC	52.27	0.0002		0.0460	0.8364	
C: DMDC	6.90	0.0340		7.43	0.0295	
AB	1.43	0.2709		0.2971	0.6027	
AC	10.29	0.0149		0.8389	0.3902	
BC	0.0756	0.7912		0.7194	0.4244	
A^2	21.62	0.0023		8.42	0.0229	
B^2	18.07	0.0038		3.81	0.0919	
C^2	26.12	0.0014		10.88	0.0131	
Lack of fit	3.79	0.1081	Not significant	3.00	0.1584	Not significant
R^2	0.9538			0.8356		
R_{adj}^2	0.8944			0.6242		

Level of significance: significant, $p < 0.05$; insignificant, $p > 0.05$.

Table S6. Results of validation experiments for BBD.

Treatment group	E2H (%)	VC (%)	DMDC (mg/L)	L* value	Firmness (N)
Control	-	-	-	58.21±0.41[d]	29.99±0.55[e]
E2H	0.010	-	-	57.57±0.36[d]	31.96±0.17[d]
VC	-	0.65	-	63.55±0.67[b]	33.51±0.26[c]
DMDC	-	-	240	59.25±0.09[c]	31.56±0.74[b]
E2H+VC+DMDC	0.010	0.65	240	66.43±0.46[a]	38.00±0.20[a]

Results are expressed as mean±SE of triplicate assays. Different letters in a column imply statistically significant differences at the $p < 0.05$ level between control and treated groups according to Duncan's multiple range test.
